# FPRs: linking innate immune system and fibrosis

**DOI:** 10.18632/oncotarget.4915

**Published:** 2015-07-18

**Authors:** Francesca Wanda Rossi, Nunzia Montuori

**Affiliations:** Department of Translational Medical Sciences and Center for Basic and Clinical Immunology Research (CISI), University of Naples Federico II, Napoli, Italy

**Keywords:** Fibrosis, fMLF receptors, systemic sclerosis, uPAR

Fibrosis is a deregulated and uncontrolled repair process that recapitulates features of embryonic development and normal wound healing. The inappropriate repair by connective tissue, characterized by an excessive deposition of collagen and other extracellular matrix components, is now known as an important feature of many chronic diseases, including myocardial infarction, glomerulosclerosis, idiopathic pulmonary fibrosis, liver cirrhosis and systemic sclerosis (SSc).

In SSc, the tightly regulated and self-limited response to injury, normally leading to tissue regeneration, is subverted into fibrosis, with disruption of tissue architecture and loss of functional integrity; both the skin and the internal organs can be affected. An inappropriate fibroblast activation and the subsequent accumulation of myofibroblasts in affected tissues underlie this switch. A subgroup of resident fibroblasts, in response to specific stimuli (e.g. transforming growth factor-β), trans-differentiate into myofibroblasts expressing high levels of α-smooth muscle actin (α-SMA) and playing a significant functional role in pathologic fibrosis [1].

*N*-formyl peptide (fMLF) receptors (FPRs) are a family of pattern recognition receptors, regulating innate responses. FPRs, by interacting with several structurally diverse pro- and anti-inflammatory ligands, possess important regulatory effects in multiple pathological conditions, including inflammation and cancer. In addition, all FPRs expressed on epithelia seem to be required for wound repair and restitution of barrier integrity, by facilitating epithelial cell migration, proliferation, and neo-angiogenesis [2].

Three variants of FPRs have been identified in humans: FPR1, FPR2, and FPR3. FPR1 is activated by nanomolar concentrations of fMLF. FPR2 is a promiscuous receptor activated in response to high concentrations of fMLF, and to viral, bacterial, endogenous and synthetic peptides. Hp(2-20), uPAR_84-95_, and F2L are natural ligands of FPR3. The synthetic peptide WKYMVm is an FPR panagonist, depending on its concentration [3].

Several functions of FPRs occur through the interaction with the urokinase-type plasminogen activator (uPA) receptor (uPAR). uPAR is formed by three homologous domains (DI, DII, DIII) anchored to the cell surface by a glycosyl-phosphatidylinositol (GPI) tail and is able to interact with integrins, FPRs and tyrosine kinase receptors, representing a main regulator of signal transduction pathways involved in wound repair, tumor progression and angiogenesis [4]. A specific region of uPAR, corresponding to amino acids 88-92 (SRSRY), located in the flexible linker connecting uPAR domains DI and DII, is able to interact with FPRs, mediating uPA or fMLF-dependent cell migration. Indeed, the uPAR-derived, synthetic uPAR_84-95_ peptide can act as a direct ligand of FPRs. uPA or its aminoterminal fragment (ATF) can promote uPAR interaction with FPRs, by determining the exposure of the uPAR_88-92_ region, upon binding to the receptor. Further, uPA-mediated removal of DI results in the membrane expression of a truncated uPAR form, that can contain the chemotactic peptide able to interact with FPRs and to regulate their signal (DII-DIII-uPAR_88-92_) [5].

**Figure:**
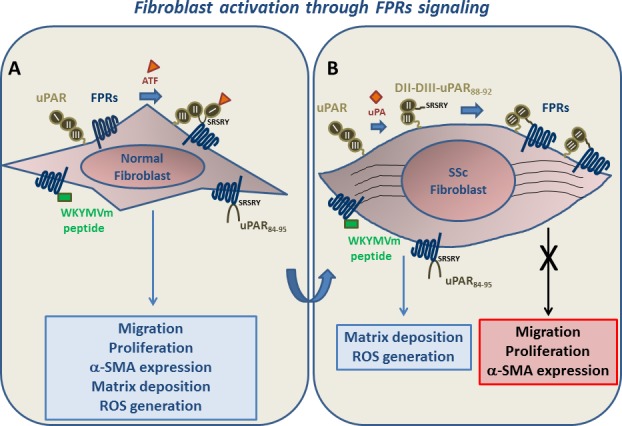
**Panel A: normal fibroblasts.** After stimulation of FPRs/uPAR cross-talk by ATF, unmasking the uPAR_88-92_ region, or FPRs engagement by WKYMVm and uPAR_84-95_ peptides, migration and proliferation increase and a myofibroblastic phenotype is acquired, through increased α-SMA expression, matrix deposition and ROS generation. **Panel B: SSc fibroblasts.** Membrane expression of FPRs and of a truncated uPAR form (DII-DIII-uPAR_88–92_), originating by uPA- or protease-mediated uPAR cleavage, is increased. DII-DIII-uPAR_88–92_ contains the SRSRY peptide that, by chronically interacting with FPRs, could desensitize them to migratory and proliferative signals. FPRs stimulation by WKYMVm and uPAR_84-95_ peptides is still able to induce matrix deposition and ROS generation.

Recently, to test the possibility of FPRs being involved in the pathogenesis of SSc, we investigated whether FPRs were expressed on human skin fibroblasts and whether their activation could play a role in some as yet unexplained processes involved in SSc, such as wound healing, tissue remodeling and fibrosis. We provided evidence, for the first time, that FPRs are expressed by normal human skin fibroblasts and that SSc fibroblasts overexpress these receptors both in *vitro* and in *vivo* [6].

FPRs could be involved in the pathogenesis of SSc through different mechanisms, including the interaction with the uPA/uPAR system. Indeed, we showed that activation of FPRs by the WKYMVm peptide and stimulation of FPRs/uPAR cross-talk by ATF and uPAR_84-95_ peptide increased proliferation and migration of normal fibroblasts and could induce a myofibroblastic phenotype, as shown by increased matrix deposition, α_v_β_5_ integrin and α-SMA expression and Radical Oxygen Species (ROS) generation. This supports the hypothesis that the SSc progressive fibrosis could be linked to the aberrant activation of FPRs signalling in fibroblasts.

uPAR deletion induces, in a murine model, pulmonary fibrosis and peripheral microvasculopathy resembling human SSc. Full-length uPAR expression is significantly downregulated in SSc dermis, especially in fibroblasts and endothelial cells [7]. We demonstrated that, in SSc fibroblasts, full length uPAR down-regulation is accompanied by increased membrane expression of DII-DIII-uPAR_88-92_. SSc fibroblasts exhibited characteristics suggesting a myofibroblast transition, such as elevated levels of α-SMA expression and vitronectin secretion, in agreement with previous studies showing that the cleavage/inactivation of uPAR is a crucial step in the fibroblast-to-myofibroblast transition [8].

We suggest that, in normal fibroblasts, innate immune signaling triggered by FPRs ligands is one of the key events converting self-limited regenerative repair into an aberrant and intractable fibrotic process, by determining increased proliferation, migration and fibroblast to myofibroblast transition. SSc fibroblasts, showing myofibroblastic features, overexpress FPRs but their proliferative and migratory signals are strongly reduced. Thus, a condition of receptor inactivation occurs, most probably through the increased membrane expression of DII-DIII-uPAR_88-92_ that, by chronically interacting with FPRs, could desensitize them. Stimulation of FPRs did not increase further α-SMA expression in SSc fibroblasts; however, their signalling was still able to promote matrix deposition, α_v_β_5_ integrin expression and ROS generation.

The capacity of FPRs, through their interaction with the uPA/uPAR system, to trigger the transformation of fibroblasts into myofibroblasts and to coordinate proliferation and cell migration makes them a potential novel therapeutic target in the treatment of early SSc.
